# Accelerating adaptive inverse distance weighting interpolation algorithm on a graphics processing unit

**DOI:** 10.1098/rsos.170436

**Published:** 2017-09-20

**Authors:** Gang Mei, Liangliang Xu, Nengxiong Xu

**Affiliations:** School of Engineering and Technology, China University of Geosciences, Beijing, People’s Republic of China

**Keywords:** geographic information system, spatial interpolation, inverse distance weighting, parallel algorithm, graphics processing unit

## Abstract

This paper focuses on designing and implementing parallel adaptive inverse distance weighting (AIDW) interpolation algorithms by using the graphics processing unit (GPU). The AIDW is an improved version of the standard IDW, which can adaptively determine the power parameter according to the data points’ spatial distribution pattern and achieve more accurate predictions than those predicted by IDW. In this paper, we first present two versions of the GPU-accelerated AIDW, i.e. the naive version without profiting from the shared memory and the tiled version taking advantage of the shared memory. We also implement the naive version and the tiled version using two data layouts, structure of arrays and array of aligned structures, on both single and double precision. We then evaluate the performance of parallel AIDW by comparing it with its corresponding serial algorithm on three different machines equipped with the GPUs GT730M, M5000 and K40c. The experimental results indicate that: (i) there is no significant difference in the computational efficiency when different data layouts are employed; (ii) the tiled version is always slightly faster than the naive version; and (iii) on single precision the achieved speed-up can be up to 763 (on the GPU M5000), while on double precision the obtained highest speed-up is 197 (on the GPU K40c). To benefit the community, all source code and testing data related to the presented parallel AIDW algorithm are publicly available.

## 1. Introduction

A spatial interpolation algorithm is the method in which the attributes at some known locations (data points) are used to predict the attributes at some unknown locations (interpolated points). Spatial interpolation algorithms, such as the inverse distance weighting (IDW) [1], Kriging [2] and discrete smooth interpolation (DSI) [3,4], are commonly used in geosciences and related research fields, especially in a geographic information system (GIS) [5]; see a brief summary in [6] and a comparative survey in [7].

Among the above-mentioned three spatial interpolation algorithms, only the Kriging method is computationally intensive due to the inversion of the coefficient matrix, while the other two are easy to compute. However, when the above three algorithms are applied to a large set of points, for example, more than 1 million points, they are still quite computationally expensive, even for the simplest interpolation algorithm IDW.

To be able to apply those interpolation algorithms in large-scale applications, the computational efficiency needs to be improved. With the rapid development of multicore central processing unit (CPU) and multicore graphics processing unit (GPU) hardware architecture, parallel computing technology has made remarkable progress. One of the most effective and commonly used strategies for enhancing the computational efficiency of interpolation algorithms is to parallelize the interpolating procedure under various massively parallel computing environments on multicore CPU and/or GPU platforms.

Since the 1990s, many researchers have devoted themselves to the parallelization of various interpolation algorithms [8–12]. Specifically for the Kriging method, many parallel programs were implemented on high-performance and distributed architectures [11,13–23]. Also, to reduce the computational cost in large-scale applications, the IDW algorithm has been parallelized under various massively parallel computing environments on multicore CPU and/or GPU platforms.

For example, by taking advantage of the power of traditional CPU-based parallel programming models, Armstrong & Marciano [8,9] implemented the IDW interpolation algorithm in parallel using Fortran 77 on shared-memory parallel supercomputers, and achieved an efficiency close to 0.9. Guan & Wu [10] performed their parallel IDW algorithms using open multi-processing (OpenMP) running on an Intel Xeon 5310, achieving an excellent efficiency of 0.92. Huang *et al.* [24] designed a parallel IDW interpolation algorithm with the message passing interface (MPI) by incorporating the message passing interface, multiple data (SPMD) and master/slave (M/S) programming modes, and attained a speed-up factor of almost 6 and an efficiency greater than 0.93 under a Linux cluster linked with six independent PCs. Li *et al.* [25] developed the parallel version of the IDW interpolation using the Java Virtual Machine (JVM) for the multi-threading functionality, and then applied it to predict the distribution of daily fine particulate matter PM 2.5.

As general purpose computing on modern GPUs can significantly reduce computational times by performing massively parallel computing, current research efforts are being devoted to parallel IDW algorithms on GPU computing architectures such as Compute Unified Device Architecture (CUDA) [26] and Open Computing Language (OpenCL) [27]. For example, Huraj *et al.* [28,29] have deployed IDW on GPUs to accelerate snow cover depth prediction. Henneböhl *et al.* [14] studied the behaviour of IDW on a single GPU depending on the number of data values, the number of prediction locations, and different ratios of data size and prediction locations. Hanzer [30] implemented the standard IDW algorithm using Thrust, PGI Accelerator and OpenCL. Xia *et al.* [31,32] developed the GPU implementations of an optimized IDW algorithm proposed by them, and obtained 13–33-fold speed-ups in computation time over the sequential version.

And quite recently, Mei [33] developed two GPU implementations of the IDW interpolation algorithm, the tiled version and the CUDA Dynamic Parallelism (CDP) version, by taking advantage of shared memory and CUDA Dynamic Parallelism, and found that the tiled version has speed-ups of 120 and 670 over the CPU version when the power parameter *p* was set to 2 and 3.0, respectively, but the CDP version is 4.8–6.0 times slower than the naive GPU version. In addition, Mei & Tian [34] compared and analysed the impact of data layouts on the efficiency of GPU-accelerated IDW implementations.

The power of GPU-based parallelization is also used in other geospatial analysis such as the viewshed analysis. For example, Xia *et al*. [31] proposed a GPU-based framework for geospatial analysis and found that the GPU implementations can lead to dataset-dependent speed-ups in the range of 28–925-fold for viewshed analysis. Strnad [35] presented the GPU-based parallel implementation of visibility calculation from multiple viewpoints on raster terrain grids. Fang *et al.* [36] presented a real-time algorithm for viewshed analysis in three-dimensional scenes by using the parallel computing capabilities of a GPU. Zhao *et al.* [37] proposed a parallel computing approach to viewshed analysis of large terrain data using GPUs.

In addition, Stojanovic & Stojanovic [38,39] developed a parallel implementation of map-matching and viewshed analysis using CUDA that was performed on contemporary GPUs. Osterman *et al.* [40] presented an IO-efficient parallel implementation of an R2 viewshed algorithm for large terrain maps on a CUDA GPU. Cauchi-Saunders & Lewis [41] presented a novel conversion of the XDraw viewshed analysis algorithm to a parallel context to increase the speed at which a viewshed can be rendered. Wang *et al.* [42] presented a real-time algorithm for viewshed analysis in 3D Digital Earth system (GeoBeans3D) using the parallel computing of GPUs.

The Adaptive Inverse Distance Weighting (AIDW) interpolation algorithm [43] is an improved version of the standard IDW. The standard IDW is relatively fast and easy to compute, and straightforward to interpret. However, in the standard IDW the distance-decay parameter is applied uniformly throughout the entire study area without considering the distribution of data within it, which leads to less accurate predictions when compared to other interpolation methods such as Kriging [43]. In the AIDW, the distance-decay parameter is a no longer constant value over the entire interpolation space, but can be adaptively calculated using a function derived from the point pattern of the neighbourhood.

The AIDW performs better than the constant parameter method in most cases, and better than ordinary Kriging in the cases when the spatial structure in the data could not be modelled effectively by typical variogram functions. In short, the standard IDW is a logical alternative to Kriging, but AIDW offers a better alternative.

As stated above, when exploited in large-scale applications, the standard IDW is in general computationally expensive. As an improved and complicated version of the standard IDW, the AIDW in this case will be also computationally expensive. To the best of the authors’ knowledge, however, there is currently no existing literature reporting the development of parallel AIDW algorithms on the GPU.

In this paper, we introduce our efforts dedicated to designing and implementing the parallel AIDW interpolation algorithm [43] on a single modern graphics processing unit (GPU). We first present a straightforward but suitable-for-paralleling method for finding the nearest points. We then develop two versions of the GPU implementations, i.e. the naive version that does not take advantage of the shared memory and the tiled version that profits from the shared memory. We also implement both the naive version and the tiled version using two data layouts to compare the efficiency. We observe that our GPU implementations can achieve satisfactory speed-ups over the corresponding CPU implementation for varied sizes of testing data.

Our contributions in this work can be summarized as follows: (i) we design the parallel AIDW interpolation algorithm by using the GPU and (ii) we develop practical GPU implementations of the parallel AIDW algorithm.

The rest of this paper is organized as follows. Section 2 gives a brief introduction to the AIDW interpolation. Section 3 introduces considerations and strategies for accelerating the AIDW interpolation and details of the GPU implementations. Section 4 presents some experimental tests that are performed on single and/or double precision. Section 5 discusses the experimental results. Finally, §6 draws some conclusions.

## 2. Background: inverse distance weighting and adaptive inverse distance weighting interpolation algorithm

### 2.1. The standard inverse distance weighting interpolation algorithm

The IDW algorithm is one of the most commonly used spatial interpolation methods in Geosciences, which calculates the prediction values of unknown points (interpolated points) by weighting the average of the values of known points (data points). The name given to this type of methods was motivated by the weighted average applied because it resorts to the inverse of the distance to each known point when calculating the weights. The difference between different forms of IDW interpolation is that they calculate the weights variously.

A general form of predicting an interpolated value *Z* at a given point *x* based on samples *Z*_*i*_=*Z*(*x*_*i*_) for *i*=1,2,…,*n* using IDW is an interpolating function:

2.1 $$Z(x)=\sum_{i=1}^{n}\frac{\omega_{i}(x)z_{i}}{\sum_{j=1}^{n}\omega_{j}(x)},\;\omega_{i}(x)=\frac{1}{d{\left(x,x_{i}\right)}^{\alpha }}.$$

The above equation is a simple IDW weighting function, as defined by Shepard [1], where *x* denotes a prediction location, *x*_*i*_ is a data point, *d* is the distance from the known data point *x*_*i*_ to the unknown interpolated point *x*, *n* is the total number of data points used in interpolating, and *p* is an arbitrary positive real number called the power parameter or the distance-decay parameter (typically, *α*=2 in the standard IDW). Note that, in the standard IDW, the power/distance-decay parameter *α* is a user-specified constant value for all unknown interpolated points.

### 2.2. The adaptive inverse distance weighting interpolation algorithm

The AIDW is an improved version of the standard IDW, which was developed by Lu & Wong [43]. The basic and most important idea behind the AIDW is that: it adaptively determines the distance-decay parameter *α* according to the spatial pattern of data points in the neighbourhood of the interpolated points. In other words, the distance-decay parameter *α* is no longer a pre-specified constant value but adaptively adjusted for a specific unknown interpolated point according to the distribution of the data points/sampled locations.

When predicting the desired values for the interpolated points using AIDW, there are typically two phases: the first one is to adaptively determine the parameter *α* according to the spatial pattern of data points; and the second is to perform the weighting average of the values of data points. The second phase is the same as that in the standard IDW; see equation (2.1).

In AIDW, for each interpolated point, the adaptive determination of the parameter *α* can be carried out in the following steps.

*Step* 1. Determine the spatial pattern by comparing the observed average nearest-neighbour distance with the expected nearest-neighbour distance.

(i) Calculate the expected nearest-neighbour distance $r_{\exp}$ for a random pattern using
  2.2 $$r_{\exp}=\frac{1}{2\sqrt{n/A}},$$
  where *n* is the number of points in the study area and *A* is the area of the study region.
(ii) Calculate the observed average nearest-neighbour distance *r*_obs_ by taking the average of the nearest-neighbour distances for all points:
  2.3 $$r_{\mathrm{obs}}=\frac{1}{k}\sum_{i=1}^{k}d_{i},$$
  where *k* is the number of nearest-neighbour points and *d*_*i*_ is the nearest-neighbour distances. The *k* can be specified before interpolating.
(iii) Obtain the nearest-neighbour statistic *R*(*S*_0_) by
  2.4 $$R(S_{0})=\frac{r_{\mathrm{obs}}}{r_{\exp}},$$
  where *S*_0_ is the location of an unknown interpolated point.

*Step* 2. Normalize the *R*(*S*_0_) measure to *μ*_*R*_ such that *μ*_*R*_ is bounded by 0 and 1 by a fuzzy membership function:

2.5 $$\mu_{R}=\left\{ \begin{array}{ll} 0 & R(S_{0})\leq R_{\min}\\ 0.5-0.5\;\cos\;\left[\frac{\pi }{R_{\max}}(R(S_{0})-R_{\min})\right] & R_{\min}\leq R(S_{0})\leq R_{\max}\\ 1 & R(S_{0})\geq R_{\max} \end{array}\right.,$$

where $R_{\min}$ or $R_{\max}$ refers to a local nearest-neighbour statistic value (in general, the $R_{\min}$ and $R_{\max}$ can be set to 0.0 and 2.0, respectively).

*Step* 3. Determine the distance-decay parameter *α* by mapping the *μ*_*R*_ value to a range of *α* by a triangular membership function that belongs to certain levels or categories of distance-decay value as follows:

2.6 $$\alpha (\mu_{R})=\left\{ \begin{array}{ll} \alpha_{1} & \text{0.0}\leq \mu_{R}\leq \text{0.1}\\ \alpha_{1}[1-5(\mu_{R}-0.1)]+5\alpha_{2}(\mu_{R}-0.1) & \text{0.1}\leq \mu_{R}\leq \text{0.3}\\ 5\alpha_{3}(\mu_{R}-0.3)+\alpha_{2}[1-5(\mu_{R}-0.3)] & \text{0.3}\leq \mu_{R}\leq 0.5\\ \alpha_{3}[1-5(\mu_{R}-0.5)]+5\alpha_{4}(\mu_{R}-0.5) & \text{0.5}\leq \mu_{R}\leq \text{0.7}\\ 5\alpha_{5}(\mu_{R}-0.7)+\alpha_{4}[1-5(\mu_{R}-0.7)] & \text{0.7}\leq \mu_{R}\leq \text{0.9}\\ \alpha_{5} & \text{0.9}\leq \mu_{R}\leq \text{1.0} \end{array}\right.,$$

where the *α*_1_, *α*_2_, *α*_3_, *α*_4_, *α*_5_ are assigned to be five levels or categories of distance-decay value.

After adaptively determining the parameter *α*, the desired prediction value for each interpolated point can be obtained via the weighting average. This phase is the same as that in the standard IDW; see equation (2.1).

## 3. Graphics processing unit-accelerated adaptive inverse distance weighting interpolation algorithm

### 3.1. Strategies and considerations for graphics processing unit acceleration

#### 3.1.1. Overall considerations

The AIDW algorithm is inherently suitable to be parallelized on GPU architecture. This is because, in AIDW, the desired prediction value for each interpolated point can be calculated independently, which means that it is natural to calculate the prediction values for many interpolated points concurrently without any data dependencies between the interpolating procedures for any pair of the interpolated points.

Owing to the inherent feature of the AIDW interpolation algorithm, it is allowed a single thread to calculate the interpolation value for an interpolated point. For example, assuming there are *n* interpolation points that are needed to be predicted their values such as elevations, and then it is needed to allocate *n* threads to concurrently calculate the desired prediction values for all those *n* interpolated points. Therefore, the AIDW method is quite suitable to be parallelized on the GPU architecture.

In GPU computing, shared memory is expected to be much faster than global memory; thus, any opportunity to replace global memory access by shared memory access should therefore be exploited [7]. A common optimization strategy is called ‘tiling’, which partitions the data stored in the global memory into subsets called tiles so that each tile fits into the shared memory [15].

This optimization strategy of ‘tiling’ is also adopted to accelerate the AIDW interpolation algorithm: the coordinates of data points are first transferred from the global memory to the shared memory; then each thread within a thread block can access the coordinates stored in the shared memory concurrently. As the shared memory residing in the GPU is limited per stream multiprocessor, the data in the global memory, that is, the coordinates of data points, need to be first split/tiled into small pieces and then transferred to the shared memory. By employing the ‘tiling’ strategy, the global memory accesses can be significantly reduced; and thus the overall computational efficiency is expected to be improved.

#### 3.1.2. Method for finding the nearest data points

The essential difference between the AIDW algorithm and the standard IDW algorithm is that: in the standard IDW, the parameter power *α* is specified to a constant value (e.g. 2 or 3.0) for all the interpolation points, while, in contrast, in the AIDW the power *α* is adaptively determined according to the distribution of the interpolated points and data points. In short, in IDW the power *α* is user-specified and constant before interpolating; but in AIDW the power *α* is no longer user-specified or constant but adaptively determined in the interpolation.

The main steps of the adaptive determination of the power *α* in the AIDW have been listed in §2.2. Among these steps, the most computationally intensive step is to find the *k* nearest neighbours (kNN) for each interpolated point. Several effective kNN algorithms have been developed by region partitioning using various data structures [21,44–46]. However, these algorithms are computationally complex in practice, and are not suitable to be used in implementing AIDW. This is because, in AIDW, the kNN search has to be executed within a single CUDA thread rather than in a thread block or grid.

**Figure 1.**
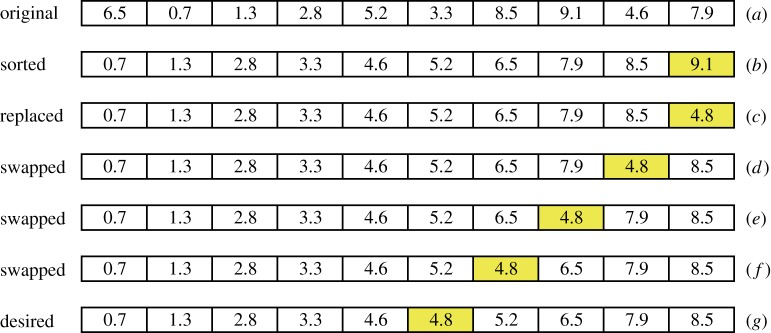
(*a*-*g*) Demonstration of the finding of *k* nearest neighbours (*k*=10).

In this paper, we present a straightforward but suitable for the GPU parallelized algorithm to find the *k* nearest data points for each interpolated point. Assuming there are *n* interpolated points and *m* data points, for each interpolated point we carry out the following steps:

*Step* 1. Calculate the first *k* distances between the first *k* data points and the interpolated points; for example, if the *k* is set to 10, then there are 10 distances that are needed to be calculated; see the row (*a*) in figure 1.

*Step* 2. Sort the first *k* distances in ascending order; see row (*b*) in figure 1.

*Step* 3. For each of the rest (*m*−*k*) data points,

(i) calculate the distance *dist*; for example, the distance is 4.8 (*dist* = 4.8);
(ii) compare the *dist* with the *k*th distance: if *dist* < the *k*th distance, then replace the *k*th distance with the *dist* (see row (*c*));
(iii) iteratively compare and swap the neighbouring two distances from the *k*th distance to the 1st distance until all the *k* distances are newly sorted in ascending order; see rows (*c*)–(*g*) in figure 1.

#### 3.1.3. The use of different data layouts

Data layout is the form in which data should be organized and accessed in memory when operating on multivalued data such as sets of three-dimensional points. The selecting of an appropriate data layout is a crucial issue in the development of GPU-accelerated applications. The efficiency performance of the same GPU application may drastically differ due to the use of different types of data layouts.

**Figure 2.**
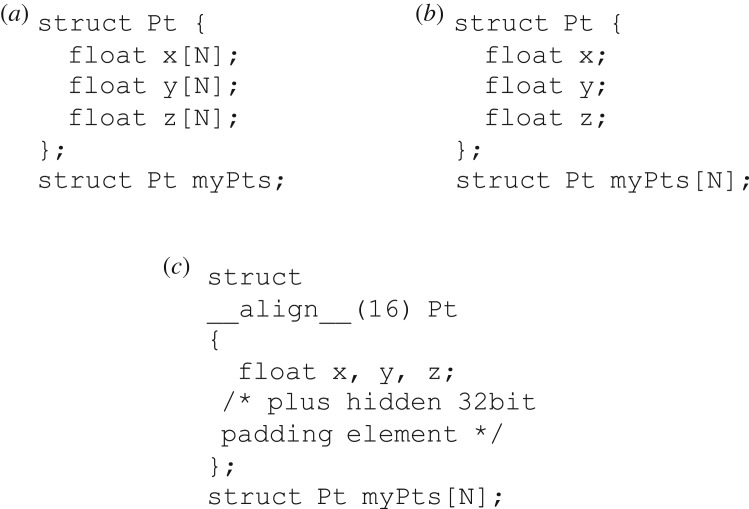
Data layouts SoA (*a*), AoS (*b*) and AoaS (*c*).

Typically, there are two major choices of the data layout: the array of structures (AoS) and the structure of arrays (SoA) [47]; another type of data layout, array of aligned structures (AoaS) [34], can be very easily generated by adding the forced alignment based on the layout AoS. In fact, the data layout AoaS can be considered as an improved variant of the layout AoS; see these three layouts, i.e. SoA, AoS and AoaS in figure 2.

Organizing data in the AoS layout leads to coalescing issues as the data are interleaved. By contrast, the organizing of data according to the SoA layout can generally make full use of the memory bandwidth due to no data interleaving [47]. In addition, global memory accesses based upon the SoA layout are always coalesced.

In practice, it is not always obvious which data layout will achieve better performance for a specific GPU application. A common solution is to implement a specific application using different data layouts separately and then compare the performances. As mentioned earlier, the data layout AoaS can be considered as an improved variant of the layout AoS; and it has been reported that the data layout AoaS can achieve better efficiency than that by the layout AoS [34]. In this work, we will evaluate the performance impact of the two data layouts, SoA and AoaS.

### 3.2. Implementation details

This section will present the details on implementing the GPU-accelerated AIDW interpolation algorithm. We have developed two versions: (i) the *naive* version that does not take advantage of the shared memory and (ii) the *tiled* version that exploits the use of the shared memory. And for both of the above two versions, two implementations are separately developed according to the two data layouts SoA and AoaS. All the source code of the presented parallel AIDW algorithm is publicly available [48].

#### 3.2.1. Naive version

In this naive version, only registers and global memory are used without profiting from the use of the shared memory. The input data and the output data, i.e. the coordinates of the data points and the interpolated points, are stored in the global memory.

Assuming that there are *m* data points used to evaluate the interpolated values for *n* prediction points, we allocate *n* threads to perform the parallelization. In other words, each thread within a grid is responsible for predicting the desired interpolation value of one interpolated point.

**Figure 3.**
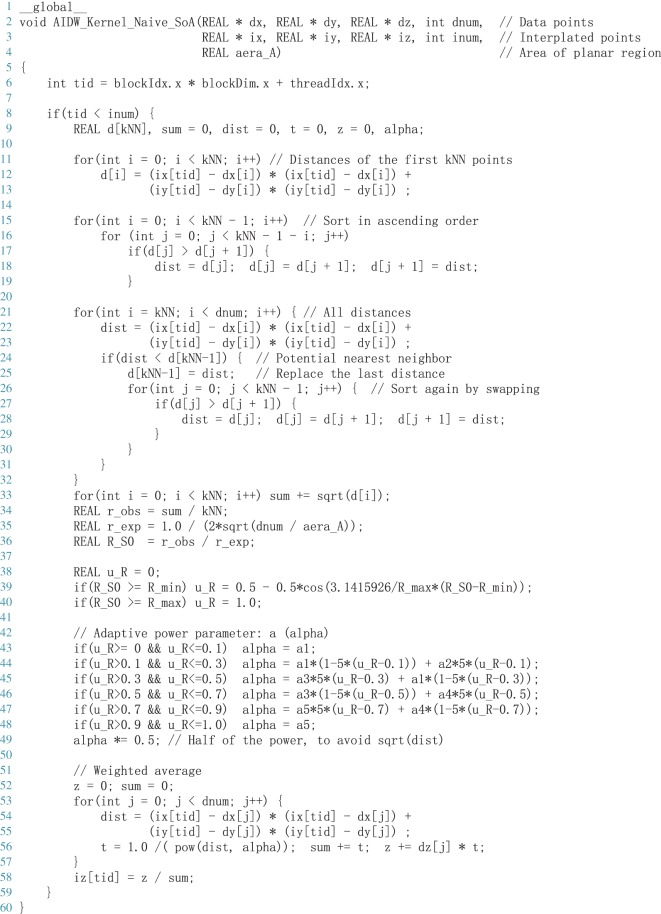
A CUDA kernel of the naive version of GPU-accelerated AIDW.

A complete CUDA kernel is listed in figure 3. The coordinates of all data points and prediction points are stored in the arrays REAL dx[dnum], dy[dnum], dz[dnum], ix[inum],iy[inum] and iz[inum]. The word REAL is defined as float and double on single and double precision, respectively.

Within each thread, we first find the *k* nearest data points to calculate the *r*_obs_ (see equation (2.3)) according to the straightforward approach introduced in §ii Method for finding the nearest data points; see the piece of code from line 11 to line 34 in figure 3; then we compute the $r_{\exp}$ and *R*(*S*_0_) according to equations (2.2) and (2.4). After that, we normalize the *R*(*S*_0_) measure to *μ*_*R*_ such that *μ*_*R*_ is bounded by 0 and 1 by a fuzzy membership function; see equation (2.5) and the code from line 38 to line 40 in figure 3. Finally, we determine the distance-decay parameter *α* by mapping the *μ*_*R*_ values to a range of *α* by a triangular membership function; see equation (2.6) and the code from line 42 to line 49.

After adaptively determining the power parameter *α*, we calculate the distances to all the data points again; and then according to the distances and the determined power parameter *α*, all the *m* weights are obtained; finally, the desired interpolation value is achieved via the weighting average. This phase of calculating the weighting average is the same as that in the standard IDW method.

Note that, in the naive version, it is needed to compute the distances from all data points to each prediction point *twice*. The first time this is carried out to find the *k* nearest neighbours/data points; see the code from line 11 to line 32; and the second is to calculate the distance-inverse weights; see the code from line 52 to line 57.

#### 3.2.2. Tiled version

The workflow of this tiled version is the same as that of the naive version. The major difference between the two versions is that, in this version, the shared memory is exploited to improve the computational efficiency. The basic ideas behind this tiled version are as follows.

The CUDA kernel presented in figure 3 is a straightforward implementation of the AIDW algorithm that does not take advantage of the shared memory. Each thread needs to read the coordinates of all data points from global memory. Thus, the coordinates of all data points are needed to be read *n* times, where *n* is the number of interpolated points.

In GPU computing, a quite commonly used optimization strategy is the ‘tiling’, which partitions the data stored in the global memory into subsets called tiles so that each tile fits into the shared memory [15]. This optimization strategy ‘tiling’ is adopted to accelerate the AIDW interpolation: the coordinates of data points are first transferred from the global memory to the shared memory; then each thread within a thread block can access the coordinates stored in shared memory concurrently.

In the tiled version, the tile size is directly set the same as the block size (i.e. the number of threads per block). Each thread within a thread block takes the responsibility of loading the coordinates of one data point from the global memory to the shared memory and then computing the distances and inverse weights for those data points stored in the current shared memory. After all threads within a block have finished computing these partial distances and weights, the next piece of data in the global memory is loaded into the shared memory and used to calculate the current wave of partial distances and weights.

It should be noted that: in the tiled version, it is needed to compute the distances from all data points to each prediction point *twice*. The first time this is carried out to find the *k* nearest neighbours/data points; and the second time is to calculate the distance-inverse weights. In this tiled version, both of the above two waves of calculating distances are optimized by employing the ‘tiling’ strategy.

By employing the ‘tiling’ strategy and exploiting the shared memory, the global memory access can be significantly reduced since the coordinates of all data points are only read (*n*/threadsPerBlock) times rather than *n* times from the global memory, where *n* is the number of prediction points and threadsPerBlock denotes the number of threads per block. Furthermore, as stated above, the ‘tiling’ strategy is applied twice.

After calculating each wave of partial distances and weights, each thread accumulates the results of all partial weights and all weighted values into two registers. Finally, the prediction value of each interpolated point can be obtained according to the sums of all partial weights and weighted values and then written into the global memory.

## 4. Results

To evaluate the performance of the GPU-accelerated AIDW method, we have carried out five groups of experimental tests on three different platforms, including one personal laptop equipped with a GeForce GT730M GPU and two workstations equipped with a Quadro M5000 GPU and a Tesla K40c GPU, respectively. All the experimental tests are run on OS Windows 7 Professional (64-bit), Visual Studio 2010 and CUDA v. 7.0. More specifications of the adopted three platforms for carrying out the experimental tests are listed in table 1.

**Table 1.** Specifications of the adopted three machines for conducting the experimental tests.

specifications	PC no. 1	PC no. 2	PC no. 3
CPU	Intel Core i7-4700MQ	Intel Xeon E5-2650 v3	Intel Xeon E5-2680 v2
CPU frequency (GHz)	2.40	2.30	2.80
CPU RAM (GB)	4	144	96
CPU core	8	40	40
GPU	GeForce GT 730M	Quadro M5000	Tesla K40c
GPU memory (GB)	1	8	12
GPU core	384	2048	2880
OS	Windows 7 Professional	Windows 7 Professional	Windows 7 Professional
compiler	Visual Studio 2010	Visual Studio 2010	Visual Studio 2010
CUDA version	v. 7.0	v. 7.0	v. 7.0

Two versions of the GPU-accelerated AIDW, i.e. the naive version and the tiled version, are implemented with the use of both the data layouts SoA and AoaS. These GPU implementations are evaluated on both single precision and double precision. However, the CPU version of the AIDW implementation is only tested on double precision; and all results of this CPU version are employed as the baseline results for comparing computational efficiency.

All the data points and interpolated/prediction points are randomly created within a square. The numbers of the prediction points and the data points are equivalent. We use the following five groups of data size, i.e. 10, 50, 100, 500 and 1000 K, where one K represents the number 1024 (1 *K*=1024).

For the GPU implementations, the recorded execution time includes the cost spent on transferring the input data point from the host to the device and transferring the output data from the device back to the host; but it does not include time consumed in creating the test data. Similarly, for the CPU implementation, the time spent for generating test data is also not considered.

### 4.1. Experiments on the PC equipped with a GeForce GT730M GPU

#### 4.1.1. Experiments on single precision

**Figure 4.**
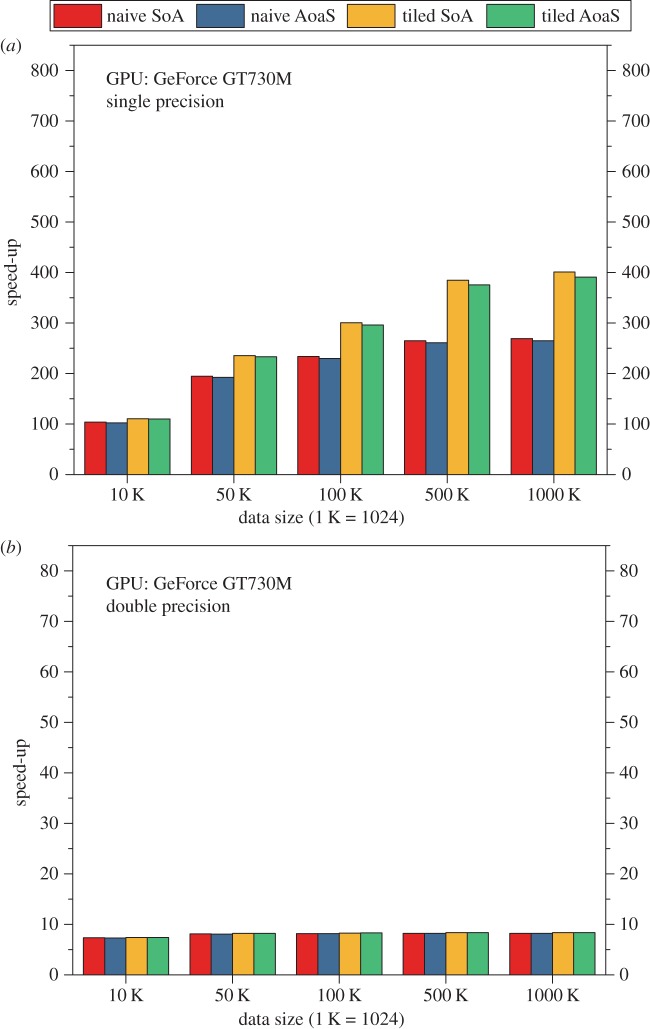
Speed-ups of the GPU-accelerated AIDW method on the GPU GT730M. (*a*) On single precision and (*b*) on double precision.

On the PC equipped with a GeForce GT730M GPU, the execution time of the CPU and GPU implementations of the AIDW on single precision is listed in table 2. And the speed-ups of the GPU implementations over the baseline CPU implementation are illustrated in figure 4*a*. According to these testing results, we have observed that:

(i) The speed-up is about 100–400; and the highest speed-up is up to 400, which is achieved by the tiled version with the use of the data layout SoA.
(ii) The tiled version is about 1.45 times faster than the naive version.
(iii) The data layout SoA is slightly faster than the layout AoaS.

**Table 2.** Execution time (ms) of CPU and GPU implementations of the AIDW method on single precision on the PC equipped with NVIDIA GPU GeForce GT730M.

		data size (1 *K*=1024)				
version	data layout	10 K	50 K	100 K	500 K	1000 K
CPU	—	6791	168 234	673 806	16 852 984	67 471 402
GPU naive	SoA	65	863	2884	63 599	250 574
	AoaS	66	875	2933	64 593	254 488
GPU tiled	SoA	61	714	2242	43 843	168 189
	AoaS	62	722	2276	44 891	172 605

In the experimental test, when the number of the data points and interpolation points is about 1 million (1000 *K*=1 024 000), the execution time of the CPU version is more than 18 h, while in contrast the tiled version only needs less than 3 min. Thus, to be used in practical applications, the tiled version of the GPU-accelerated AIDW method on single precision is strongly recommended.

#### 4.1.2. Experiments on double precision

We also evaluate the computational efficiency of the naive version and the tiled version on double precision (table 3). It is widely known that the arithmetic operated on the GPU architecture on double precision is inherently much slower than that on single precision. In our experimental tests, we also clearly observed this behaviour: on double precision, the speed-up of the GPU version over the CPU version is only about 8 (figure 4*b*), which is much lower than that achieved on single precision.

**Table 3.** Execution time (ms) of CPU and GPU implementations of the AIDW method on double precision on the PC equipped with NVIDIA GPU GeForce GT730M.

		data size (1 *K*=1024)				
version	data layout	10 K	50 K	100 K	500 K	1000 K
CPU	—	6791	168 234	673 806	16 852 984	67 471 402
GPU naive	SoA	924	20 761	82 400	2 047 590	8 184 090
	AoaS	929	20 821	82 524	2 050 269	8 199 389
GPU tiled	SoA	915	20 521	81 306	2 017 650	8 062 332
	AoaS	916	20 505	81 218	2 016 367	8 057 219

We have also observed that: (i) there are no performance gains obtained from the tiled version against the naive version and (ii) the use of data layouts, i.e. SoA and AoaS, does not lead to significant differences in computational efficiency.

As observed in our experimental tests, on double precision the speed-up generated in most cases is approximately 8, which means the GPU implementations of the AIDW method are far from practical usage. Thus, we strongly recommend users to prefer the GPU implementations on single precision for practical applications.

### 4.2. Experiments on the PC equipped with a Quadro M5000 GPU

#### 4.2.1. Experiments on single precision

**Figure 5.**
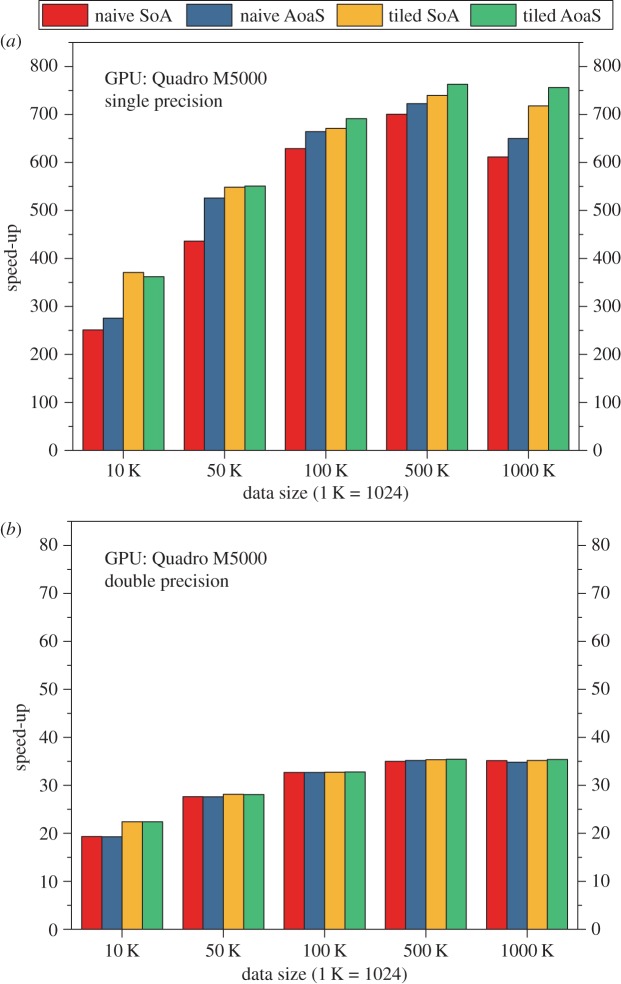
Speed-ups of the GPU-accelerated AIDW method on the GPU M5000. (*a*) On single precision and (*b*) on double precision.

On the PC equipped with a Quadro M5000 GPU, the execution time of the CPU and GPU implementations of the AIDW on single precision is listed in table 4. The speed-ups of the GPU implementations over the baseline CPU implementation are illustrated in figure 5*a*. According to these testing results, we have observed that:

(i) The speed-up is about 250–750; and the highest speed-up is up to 763, which is achieved by the tiled version with the use of the data layout AoaS.
(ii) The tiled version is about 1.16 times faster than the naive version.
(iii) The data layout AoaS is slightly faster than the layout SoA.

**Table 4.** Execution time (ms) of CPU and GPU implementations of the AIDW method on single precision on the PC equipped with NVIDIA GPU Quadro M5000.

		data size (1 *K*=1024)				
version	data layout	10 K	50 K	100 K	500 K	1000 K
CPU	—	5897	141 582	576 228	14 224 126	57 207 987
GPU naive	SoA	24	325	917	20 307	93 556
	AoaS	21	269	867	19 685	87 992
GPU tiled	SoA	16	258	859	19 225	79 692
	AoaS	16	257	833	18 640	75 646

#### 4.2.2. Experiments on double precision

We also evaluate the computational efficiency of the naive version and the tiled version on double precision (table 5). In our experimental tests, we have clearly observed that, on double precision, the speed-up of the GPU version over the CPU version is about 20–35 (figure 5*b*), which is much lower than that achieved on single precision.

**Table 5.** Execution time (ms) of CPU and GPU implementations of the AIDW method on double precision on the PC equipped with NVIDIA GPU Quadro M5000.

		data size (1 *K*=1024)				
version	data layout	10 K	50 K	100 K	500 K	1000 K
CPU	—	5897	141 582	576 228	14 224 126	57 207 987
GPU naive	SoA	305	5121	17 626	406 338	1 628 726
	AoaS	306	5127	17 635	404 180	1 643 925
GPU tiled	SoA	263	5038	17 589	402 488	1 626 039
	AoaS	263	5040	17 587	401 551	1 616 581

As is the case with the GPU GT730m, we have also observed that: (i) there are no performance gains obtained from the tiled version against the naive version and (ii) the use of data layouts, i.e. SoA and AoaS, does not lead to significant differences in computational efficiency.

### 4.3. Experiments on the PC equipped with a Tesla K40c GPU

#### 4.3.1. Experiments on single precision

**Figure 6.**
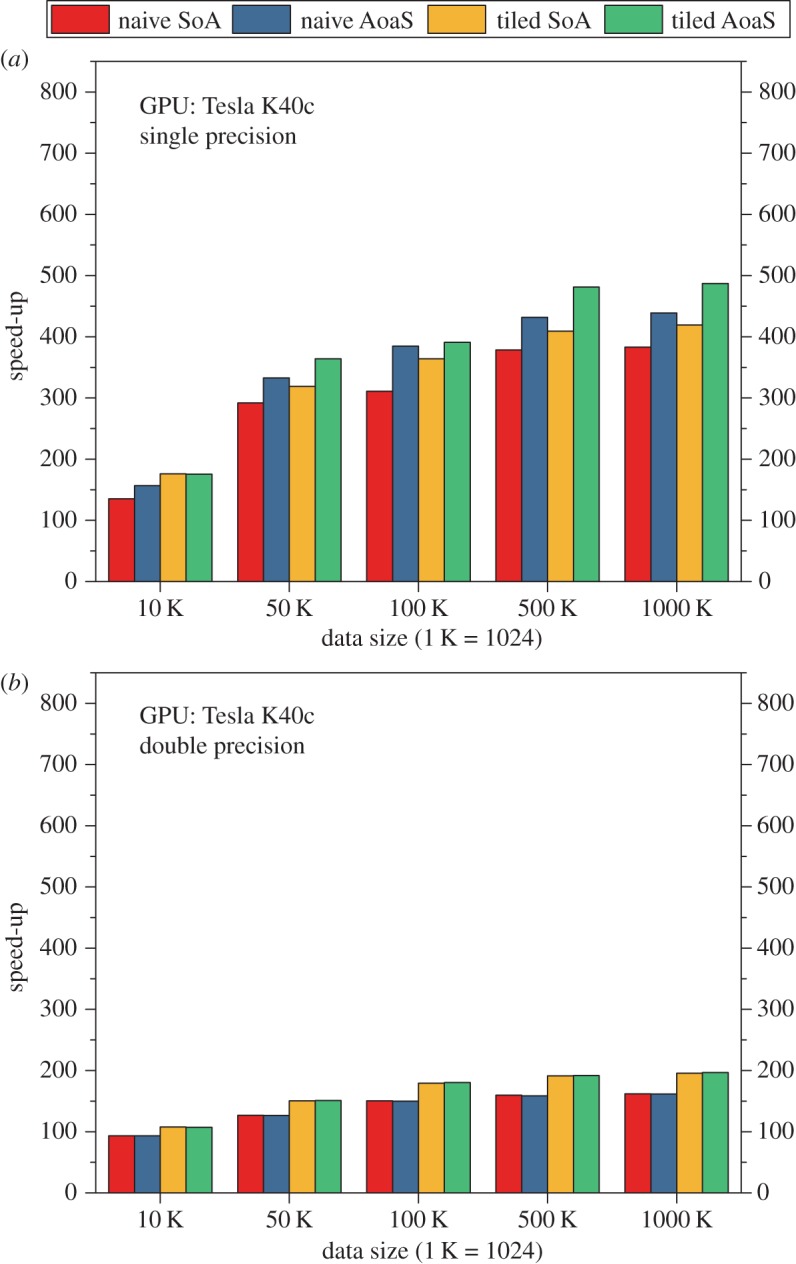
Speed-ups of the GPU-accelerated AIDW method on the GPU K40c. (*a*) On single precision and (*b*) on double precision.

On the PC equipped with a Tesla K40c GPU, the execution time of the CPU and GPU implementations of the AIDW on single precision is listed in table 6. The speed-ups of the GPU implementations over the baseline CPU implementation are illustrated in figure 6*a*. According to these testing results, we have observed that:

(i) The speed-up is about 130–480; and the highest speed-up is up to 487, which is achieved by the tiled version with the use of the data layout AoaS.
(ii) The tiled version is about 1.10 times faster than the naive version.
(iii) The data layout AoaS is slightly faster than the layout SoA.

**Table 6.** Execution time (ms) of CPU and GPU implementations of the AIDW method on single precision on the PC equipped with NVIDIA GPU Tesla K40c.

		data size (1 *K*=1024)				
version	data layout	10 K	50 K	100 K	500 K	1000 K
CPU	—	5195	118 576	475 270	11 793 605	47 368 231
GPU naive	SoA	39	406	1527	31 166	123 601
	AoaS	33	356	1236	27 314	107 927
GPU tiled	SoA	30	372	1307	28 835	112 968
	AoaS	30	326	1216	24 518	97 235

#### 4.3.2. Experiments on double precision

We also evaluate the computational efficiency of the naive version and the tiled version on double precision (table 7). The speed-up of the GPU version over the CPU version is about 100–200 (figure 6*b*), which is much lower than that achieved on single precision.

**Table 7.** Execution time (ms) of CPU and GPU implementations of the AIDW method on double precision on the PC equipped with NVIDIA GPU Tesla K40c.

		data size (1 *K*=1024)				
version	data layout	10 K	50 K	100 K	500 K	1000 K
CPU	—	5195	118 576	475 270	11 793 605	47 368 231
PU naive	SoA	56	934	3156	73 818	292 422
	AoaS	56	936	3166	74 268	293 565
GPU tiled	SoA	48	789	2649	61 640	242 046
	AoaS	48	786	2639	61 416	240 968

As with the GPUs GT730M and M5000, we have also observed that: (i) there are no performance gains obtained from the tiled version against the naive version and (ii) the use of data layouts, i.e. SoA and AoaS, does not lead to significant differences in computational efficiency.

## 5. Discussion

### 5.1. Impact of data layout on the computational efficiency

**Figure 7.**
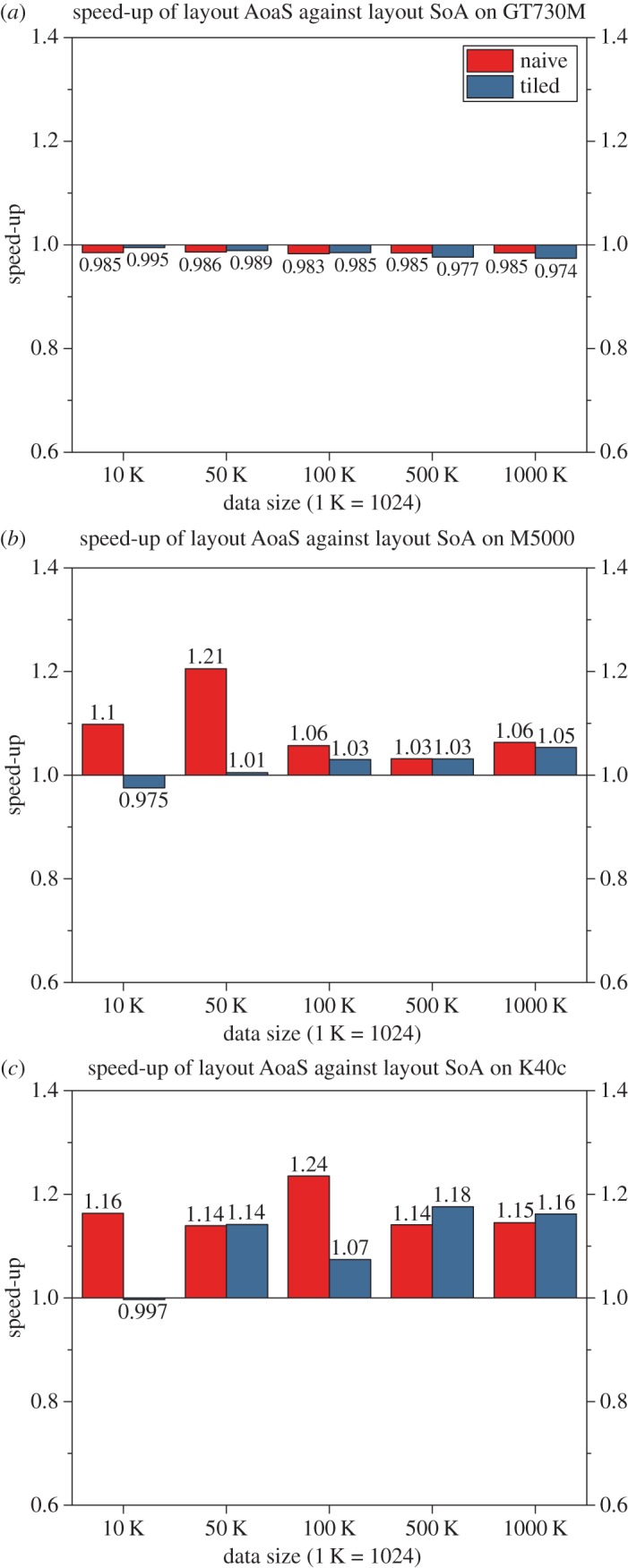
Performance comparison of the layouts SoA and AoaS.

In this work, we have implemented the naive version and the tiled version with the use of two data layouts SoA and AoaS. In our experimental tests, we have found that, on single precision, the SoA data layout can achieve a slightly better efficiency than the AoaS on the GPU GT730M, while the data layout AoaS is slightly better than the SoA on both GPUs M5000 and K40c. However, there is no significant difference in the efficiency when using the above two data layouts on the adopted three GPUs (figure 7).

Each type of data layout has its theoretical advantages. Theoretically, organizing data in the SoA layout can generally make full use of the memory bandwidth due to no data interleaving [47]. In addition, global memory accesses based upon the SoA layout are always coalesced. By contrast, when employing the AoaS layout, the data structure for representing multivalued data such as a set of three-dimensional points is forced to be aligned (figure 2). Operations using the aligned structure would require much fewer memory transactions when accessing global memory, and thus the overall performance efficiency is increased [26].

In practice, the impact of data layouts on computational efficiency strongly depends on: (i) the particular problem that needs to be solved and (ii) the GPUs used to deal with the target problem.

In this work, the experimental tests indicate that: on single precision, the SoA data layout can achieve slightly better efficiency than the AoaS on the GPU GT730M, while the data layout AoaS is slightly better than the SoA on both the GPUs M5000 and K40c. The reason for the above behaviour is probably that: for the same specific application, the impact of data layouts on computational efficiency may differ on different GPUs.

Although the two data layouts lead to different impacts on computational efficiency, there is no significant difference in the efficiency when using the above two data layouts on the adopted three GPUs (figure 7). This is probably because the AIDW interpolation algorithm is quite suitable to be parallelized on the GPU, and the parallelization has been well implemented. Therefore, the impact of different data layouts on efficiency for a specific highly parallelized application would not be significant.

In summary, the impact of data layouts on computational efficiency may differ on different GPUs. It also should be noted that, it is not always obvious which data layout will achieve better performance for a specific application.

### 5.2. Performance comparison of the naive version and tiled version

**Figure 8.**
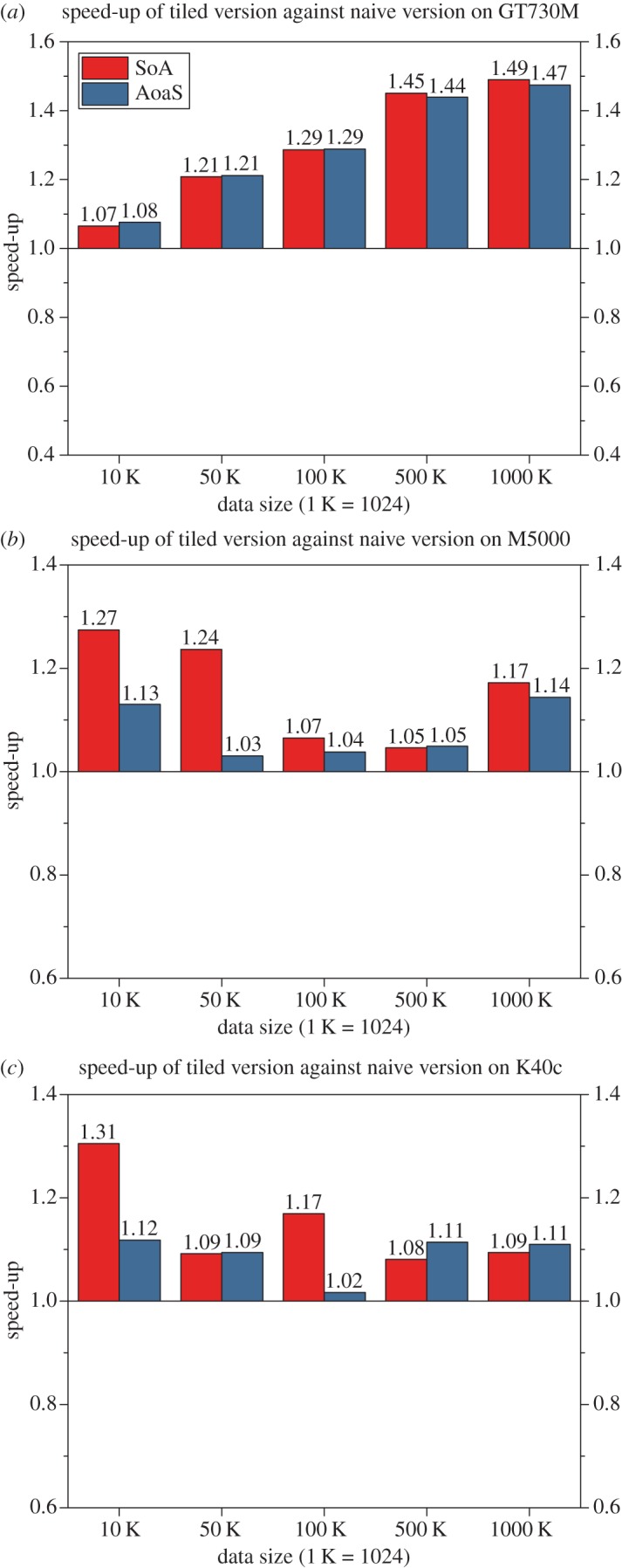
Performance comparison of the naive version and tiled version.

In our experimental tests, we also observed that the tiled version is always a little faster than the naive version no matter which data layout is adopted; see figure 8. This performance gain is due to the use of the shared memory according to the optimization strategy of ‘tiling’.

On the GPU architecture, the shared memory is inherently much faster than the global memory; thus any opportunity to replace global memory access by shared memory access should therefore be exploited.

In the tiled version, the coordinates of data points originally stored in the global memory are divided into small pieces/tiles that fit the size of shared memory, and then loaded from slow global memory to fast shared memory. These coordinates stored in the shared memory can be accessed quite fast by all threads within a thread block when calculating the distances. By blocking the computation in this way, we take advantage of fast shared memory and significantly reduce global memory accesses: the coordinates of data points are only read (*n*/threadsPerBlock) times from the global memory, where *n* is the number of prediction points.

This is the reason why the tiled version is faster than the naive version. Therefore, from the perspective of practical usage, we recommend the users to adopt the tiled version of the GPU implementations.

### 5.3. Performance comparison on single precision and double precision

Unlike the computations on the CPU, the computational efficiency on the GPU architecture is significantly varied on different precisions (e.g. on single precision and double precision). More specifically, computations on single precision are dramatically faster than those on double precision. This behaviour is inherent on GPUs.

One of the key reasons for this has been revealed in the CUDA Programming Guide [26]. The processing power of the GPU is highly determined by the number of the residing warps on each multiprocessor for a given kernel; and the number of registers used by a kernel can have a significant impact on the number of resident warps. Each double variable uses two registers, while each float variable only needs one register. In this case, for a specific kernel, typically the number of registers used by the kernel on double precision is much larger than that on single precision. Thus, the number of resident warps on each multiprocessor reduces, and the computational efficiency decreases.

The above behaviour has also been clearly observed in our experimental results. On the three GPUs used for conducting the experimental tests, the calculations on single precision are approximately 14–48, 13–21 and 1.5–2.5 times faster than those on double precision on the GPUs GT730M, M5000 and K40c, respectively.

In more detail, on the three GPUs GT730M, M5000 and K40c, the achieved speed-ups on single precision are about 100–400, 250–750 and 130–480 while in contrast the speed-ups are approximately 8, 20–35 and 100–200 on double precision (figures 4–6).

According to these experimental results, the tiled version of the GPU-accelerated AIDW method on single precision is strongly recommended in practical applications.

Moreover, in various scientific computations, if the computation operated on single precision reaches the requirement of computational accuracy, then single precision should be preferred. By contrast, if high computational efficiency is required, then (i) double precision is needed to be used and (ii) a suitable GPU that can well support computations on double precision is also required, for example, the Tesla K40c GPU in our experimental tests.

## 6. Conclusion

In this paper, we have developed two versions of the parallel AIDW interpolation algorithm by using a single GPU, i.e. the naive version that does not profit from shared memory and the tiled version that takes advantage of shared memory. We have also implemented the naive version and the tiled version with the use of two data layouts, AoS and AoaS, on both single precision and double precision. We have evaluated the computational performance of the presented parallel AIDW algorithm on three different GPUs (i.e. GT730M, M5000 and K40c). We have observed that: (i) there is no significant difference in the computational efficiency when different data layouts are employed; (ii) the tiled version is always slightly faster than the naive version and (iii) on single precision the achieved speed-up can be up to 763 (on the GPU M5000), while on double precision the obtained highest speed-up is 197 (on the GPU K40c). To benefit the community, all source code and testing data related to the presented AIDW algorithm are publicly available [48].

## Data accessibility

The source code and sample testing data have been deposited in Dryad at http://dx.doi.org/10.5061/dryad.ck2br [48].
